# Characteristics of Ultrasound and Magnetic Resonance Imaging of Normal Testes and Epididymis Besides Angiography of Testicular Artery in Dromedary Camel

**DOI:** 10.3389/fvets.2022.899570

**Published:** 2022-06-22

**Authors:** Ramadan Sary, Karim Khalil, Ramya A. Sindi, Ragab H. Mohamed, Hassan A. Hussein, Refaat A. Eid, Haney Samir, Mohammed M. Alkahtani, Ayman A. Swelum, Ahmed E. Ahmed

**Affiliations:** ^1^Department of Anatomy and Embryology, Faculty of Veterinary Medicine, Cairo University, Giza, Egypt; ^2^Department of Veterinary Medicine, College of Applied and Health Sciences, A'Sharqiyah University, Ibra, Oman; ^3^Department of Laboratory Medicine, Faculty of Applied Medical Sciences, Umm Al-Qura University, Mecca, Saudi Arabia; ^4^Department of Theriogenology, Faculty of Veterinary Medicine, Aswan University, Aswan, Egypt; ^5^Department of Theriogenology, Faculty of Veterinary Medicine, Assiut University, Assiut, Egypt; ^6^Department of Pathology, College of Medicine, King Khalid University, Abha, Saudi Arabia; ^7^Department of Theriogenology, Faculty of Veterinary Medicine, Cairo University, Giza, Egypt; ^8^Department of Biology, College of Science, King Khalid University, Abha, Saudi Arabia; ^9^Department of Theriogenology, Faculty of Veterinary Medicine, Zagazig University, Zagazig, Egypt; ^10^Department of Theriogenology, Faculty of Veterinary Medicine, South Valley University, Qena, Egypt

**Keywords:** sonography, MRI, dromedary camel, breeding season, reproduction

## Abstract

Decreasing male fertility encouraged the investigators to innovate accurate diagnostic non-invasive methods for detection of changes in the testicular parenchyma. Ultrasonography (US) has the potential to be used in this manner for decades, but magnetic resonance imaging (MRI) is still of limited application in animals for this purpose. The current study was designed to describe appearances and quantitative MRI attributes of the normal testes, epididymis besides angiography of testicular artery in camels. About 30 apparently healthy male dromedary camels aged 8–14 years were slaughtered during the rutting season. Immediately after slaughtering, the male gonads (*n* = 30 pairs of testicles and epididymis) were subjected to morphometric evaluation using a Vernier caliper and ultrasound scanning. Epididymial sperms were evaluated for motility, vitality and abnormality. MRI was performed for testes (*n*=16) by using a 1.5T Excite-II MRI apparatus of Sigma. Radiography and angioarchitecture of testicular artery (*n*=24) were done. Camel testicular length, width, and depth showed non-significant differences between a Vernier caliper or sonar. The MRI results revealed that both the testis and epididymis have homogenously intermediate signal (T1) and testes have hyperintense signal, with slightly lower signal in the epididymis (T2). In conclusion, both the ultrasonography and MRI techniques, with each respective computer-assisted imaging, could be used to detect the histomorphological changes of the camels' testicles. However, US imaging remains the first diagnostic technique for evaluating the reproductive health in men for its lower cost and accuracy. MRI is beneficial when the sonograms are inconclusive and/or equivocal. It shows the examined tissues in greater anatomical details compared to ultrasonography. Further studies are needed to compare between characteristics of US and MRI of normal testes and epididymis with testicular artery angiography in living camel during rut season and non-rut season and between normal healthy and affected diseased genitalia.

## Introduction

Arabian countries raise more than 70% of total population of dromedary camels (*C. dromedarius*) (1). The camel is used for production of milk, meat, hair, and wool as well as for draught and riding purposes as culture and traditions in Arabian deserts with ability to survive under harsh conditions (2, 3). However, low reproductive performance is the main factor of inferior productivity of camels. Several hereditary or environmental factors are implicated in the lowered fertility of camels (4–6). Understanding the reproductive anatomy improves the pattern, efficiency and performance of using the assisted reproductive technologies in the camel (7).

The clinical testicular pathology cases can be diagnosed through the apparent and unapparent symptoms. Methods monitoring male fertility, like; testicle biopsies and testosterone assays are invasive, stressful, and expensive (8). Semen evaluation exhibits significant circannual varieties but couldn't indicate upcoming reproductive potential in camel (9), ram (10), and human (8, 11). Furthermore, assessment of camel semen can't frequently be carried out since the repeated emissions of semen could alter the features of the ejaculated spermatozoa and its abundance (10). Semen collection from the aggressive camels is very difficult in the rutting season; moreover, the camel semen with its viscous nature hinders their evaluation and processing (12, 13). Two technologies showing the ability to monitor the histomorphological dynamics in testicular tissue in accurate and non-invasive manner; the ultrasonography (US) and magnetic resonance imaging (MRI). In some cases, as testicular tumors and infarction, the clinical diagnosis is inconclusive without the imaging techniques, i.e., US and MRI.

Recently, ultrasound scanning using high frequency probes is considered as a supplementary tool for clinical diagnosis of testicular diseases. Ultrasonography can provide valuable information on the normal anatomy, morphological features and vascularity of the examined organ. Moreover, US is performed without ionizing radiation and patient discomfort. It can detect the major apparent changes regarding the tissue density, molecular composition, pixels' intensity (brightness) and frequency of the sound beaming (14, 15).

Nevertheless, US fails to clinically diagnose some cases. US is frequently used in the diagnosis of testicular or scrotal masses, while the MRI serves as a confirmative modality when the US fails to establish an accurate diagnosis (16). Detailed images with high resolution of testes could be obtained by MRI. The multiplanar capabilities combined with high resolution can provide valuable diagnosis. It provides a high magnetic field (B0) aligning the magnetic hydrogen atoms in a low energy in the tissue configuring spins resulting in a high spatial resolution with a clearer contrast of gray to white matter compared to that is used in X-ray (17). MRI is an accurate imaging technique visualizing the internal structures containing water, lipids and proteinsby using the minimal magnetics found around the abundant hydrogen atoms' protons in these tissues (18, 19).

Recently, MRI thoroughly evaluates the normal bones and joints tissues of a dromedary camel (20, 21). MRI appearances of normal testes must be considered before evaluation of the pathologically diagnosed cases. To the best of our knowledge, MRI appearances of the normal camel's testes and epididymis have not yet been described. MRI-produced imaging quality is known as better than those produced by US considering the low signal-noise ratio in the later. However, the reduced background noise allows an accurate quantification of magnetic resonance parameters (18, 22). MRI efficacy is higher than US efficacy in detecting the testicular pathologies, such as; germ cell tumor and fibrosis (23). Our study aimed to: (a) record attributes of US and MRI abilities in determining the characteristics of normal camel testes ex-situ, and (b) describes the morphometric and angiographic appearance of the associated testicular artery.

## Materials and Methods

### Male Gonads Collection

Thirty male dromedary camels which were apparently healthy and aged 8–14 years, during the rutting season period from December to April, were used. Immediately after slaughtering the animals in the local abattoir, the testicles (*n* = 30 pairs) were obtained in normal saline and transported to our laboratory within 1 h for processing and investigations.

### Morphometric Evaluation of Testes and Associated Epididymides

The testicular measurements, such as testicular width, length, and depth, were taken using a caliper at their maximum points per testis for all samples (60 testes). Heads and tails of the epididymides were measured using a Vernier caliper. Testicular volume was calculated as: volume = [length × width × height] ×0.5236 (24, 25).

### Ultrasonographic Examination

Ultrasound scanning was performed by using portable ultrasound apparatus (MyLabTM 30VETGold; Esaote Italy) with a 6/8-MHz convex transducer (MyLabTM; Model AC2541 VET; Esaote) through degassed water bath (38°C). The organ was longitudinally and transversely scanned. Thicknesses of the tunica albuginea and mediastinum testis were evaluated from the longitudinal plane in both sagittal and cross-sections. Caput epididymis was diagonally scanned near the ventral end of the testis.

### Evaluation of Epididymal Spermatozoa

The epididymal spermatozoa were evaluated to confirm the fertility of the camels. A total of 20 epididymis was dissected and their tails were incised longitudinally and rinsed 3–4 times using Brackett and Oliphant (BO) medium in Petri dishes of 60-mm size (Liverpool, Australia, Bacto Lab.) placed on a warm stage (37°C) to obtain a sperm-rich fluid (24). For evaluating the epididymal spermatozoa, the freshly collected spermatozoa were examined for the mass and progressive motilities using the phase-contrast microscopy. The percentages of sperm vitality and abnormality were determined from fixed-smear stained with eosin-nigrosin. Briefly, on a preheated (37°C) clean and dry slide, 1 drop of semen was gently homogenized and mixed with 2 drops of red eosin and 4 drops of the black nigrosin stain. Then, thin smears were prepared from the mixture and examined at a 40 × magnification of the light microscopy. A totally of 300 spermatozoa were examined in the whole stained smears' fields showing; colored dead sperm vs. colorless live sperm cells. Sperm morphology was categorized into normal or abnormal (25). Sperm motility and vitality were >60 %; while sperm abnormality was <30 %.

### Imaging With Magnetic Resonance (MRI)

**Figure 1:**
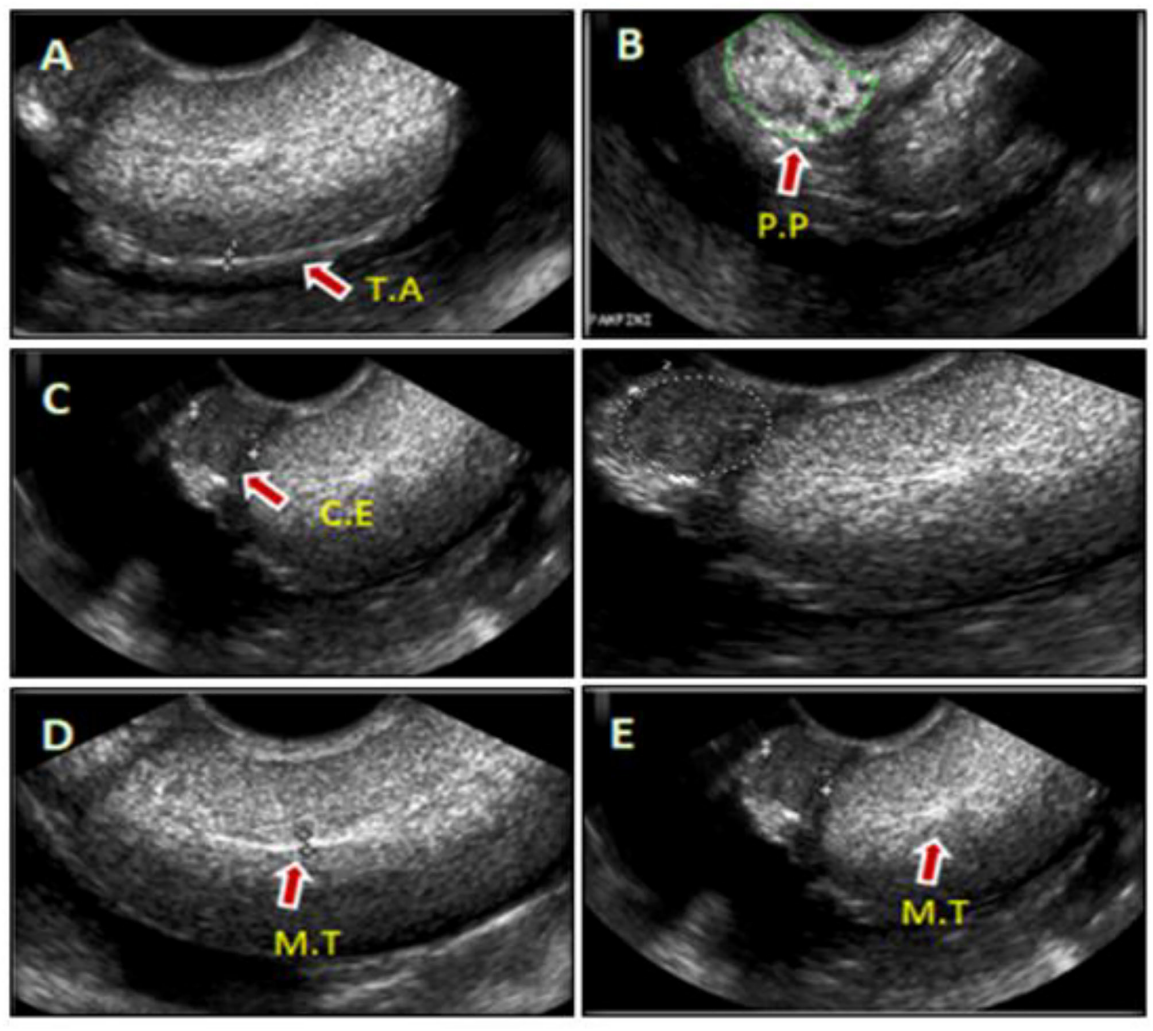
Representative images of camel's testis and cauda epididymal parenchyma scanned by the ultrasound (US) during rut season **(A–E)**. The sonograms were shown in the sagittal or cross-sections of testis and that associated epididymis, such as; tunica albuginea (T.A) **(A)**, pampiniform plexus (P.P) **(B)**, cauda epididymis (C.E) **(C)**, and mediastinum of the testis (M.T) **(D,E)**.

Immediately following the US, MRI was performed for testes (*n* = 16) by using a 1.5T Excite-II MRI apparatus of Sigma (General Electric Med. Systems, Milwaukee, WI, USA; V.11). As previously described (26), the testicles were kept in moist state and arranged to match those scanned by US (Figure 1). Four MRI scans were used; (a) 3-plane localizer; 3-plane loc., a quick scan acquiring three planes a scan, no need to additional scanning, with 1.5 mm spacing (SP) of consecutive frames, and 3.0 spatial resolution (THK) were applied; (b) a fact-sensitive series measuring longitudinal relaxation time (T1) of protons; T1-Spin Echo (T1SE), THK/SP=2.0/0.0 mm; (c) a water-sensitive series measuring the transverse relaxation time (T2) of protons; T2-Fast Spin Echo (T2FSE), THK/SP = 2.0/0.0 mm; and d) a 3-D series using a fast spoiled gradient recall pulse sequence to obtain a 3-D image with good resolution; 3-D Fast-Spoiled Gradient Echo (3-D FSPGRE), THK/SP=1.0/0.0 mm. Appearance of T1SE and T2FSEMR images were variant from each other due to arrangement of protons in the various tissue areas; those with long T1 time and short T2 time, as water, appeared dark in the T1SE-MR images and bright in the T2FSE-MR images. Tissues with short T1- and long T2-time, as fat, appeared bright vs. dark in the T1SE and T2FSE images, respectively (Figure 1). The images of MRI were produced by Centricity DICOM viewer (General Electric Medical Systems, USA; V3.1.2;). One image per testis was selected to represent the scanning modality, with four selected images, as a total, and saved as a JPEG format. The same 3-points of interest were used for image analysis as explained for ultrasound imaging.

### Radiography and Angioarchitecture of Testicular Artery

After ultrasonographic measurements, the collected camels' testicles (*n* = 24) were prepared for injection of the testicular arteries with epoxy latex (Urographine^®^). The testes were immersed into normal saline at ambient air then divided into three groups, in all of which the testicular artery was cannulated at the apical part vascular cone to primarily be thoroughly washed using heparin (5,000 I.U.)-containing saline for getting rid of any blood clots. The 1^st^ group; 10 testicles were injected with latex, Urographine, and then imaged in three ways: anteroposterior, mediolateral up to oblique radiographs. A 75 cm FFD, with 40KVP and 100 MAS were the exposure factors. The 2nd group, another 10-specimens were treated with injection of gum milk latex colored with red Rotering^®^ ink. Thereafter, the specimens were only kept into 10% formalin for 8 days before dissection of the arterial loops. The last 3rd group, ten specimens were assigned for a corrosive cast technique using Epoxy; E-151N20. After hardening for 1–2 weeks at the room temperature, they were corroded into HCL for a day, then washed by running water and allowed to dry.

The abdominal part of testicular artery was traced for course and origin in the slaughterhouse. Both length and width of vessels were measured by using the Vernier caliper supported by the magnifying lens. The method used for detecting the degrees of convolution was applied by counting the loops found in the testicular artery along its course. All specimens were saved with nomenclature by using a digital photography for documentation (27).

### Statistical Analysis

All data were statistically analyzed using ANOVA, by the GraphPad Prism Software (San-Diego, CA, USA; V.3). All values were presented as mean ± SEM. Tukey was used as a *post-hoc* for confirming the significance between differences of treatments, and finally distinguished by the Duncan's multiple pattern. The significance was considered when *p* < 0.05.

## Results

### Camel Testes and Epididymis Morphometry Using Caliber and Ultrasonography During Rut Season

Characteristics of the camel testicles were shown in Table 1. Camel testicular length, width and depth showed non-significant differences between the testes examined by caliper and sonar, either. However, the left testis' morphometric parameters were significantly higher (*p* ≤ 0.05) than each respective parameter of the other right ones (Table 1).

**Table 1:** Camel testicular measurements by using the caliper and ultrasound scanning.

Parameters*	**Left testes**			**Right testes (*****n*** **=** **30)**		
	**Vernier caliper (*n*=30)**	**Ultrasound caliper (*n* = 30)**	**MRI caliper (*n* = 8)**	**Vernier caliper (*n* = 30)**	**Ultrasound caliper (*n* = 30)**	**MRI caliper (*n* = 8)**
Testicular width	4.13 ± 0.03	3.84 ± 0.01	3.85 ± 0.01	3.82 ± 0.03	3.71 ± 0.01	3.73 ± 0.01
Testicular length	7.22 ± 0.24	7.13 ± 0.23	7.12 ± 0.21	6.54 ± 0.35	6.51 ± 0.33	6.50 ± 0.32
Testicular depth	3.62 ± 0.02	3.61 ± 0.01	3.60 ± 0.01	3.55 ± 0.02	3.53 ± 0.02	3.54 ± 0.02
Testicular volume	55.64 ± 1.24	51.57 ± 1.25	51.50 ± 1.24	45.27 ± 1.21	44.37 ± 1.24	44.07 ± 1.25

^*^There is no significant difference between Vernier caliper, Ultrasound and MRI in all measured parameters.

*The left testis' morphometric parameters were significantly higher (p ≤ 0.05) than each respective parameter of the other right ones*.

Ultrasonographic scanning showed that the testicular tunics appeared as hyperechoic membranes with a diameter of 2.0 ± 0.04 mm surrounding a homogenous hyperechoic testicular parenchyma and the hyperechogenicity appeared moderate toward the end of examination period (April) (Figure 1A). Pampiniform plexus and cauda epididymis were clearly visualized in the ultrasound cross-sections (Figures 1B,C, respectively). The testicular mediastinum appeared as a central hyperechogenicline of sagittal sections (Figure 1D), but a spot-like in the cross-sections (Figure 1E).

### Magnetic Resonance Imaging of Camel Testes, and Epididymides

**Figure 2:**
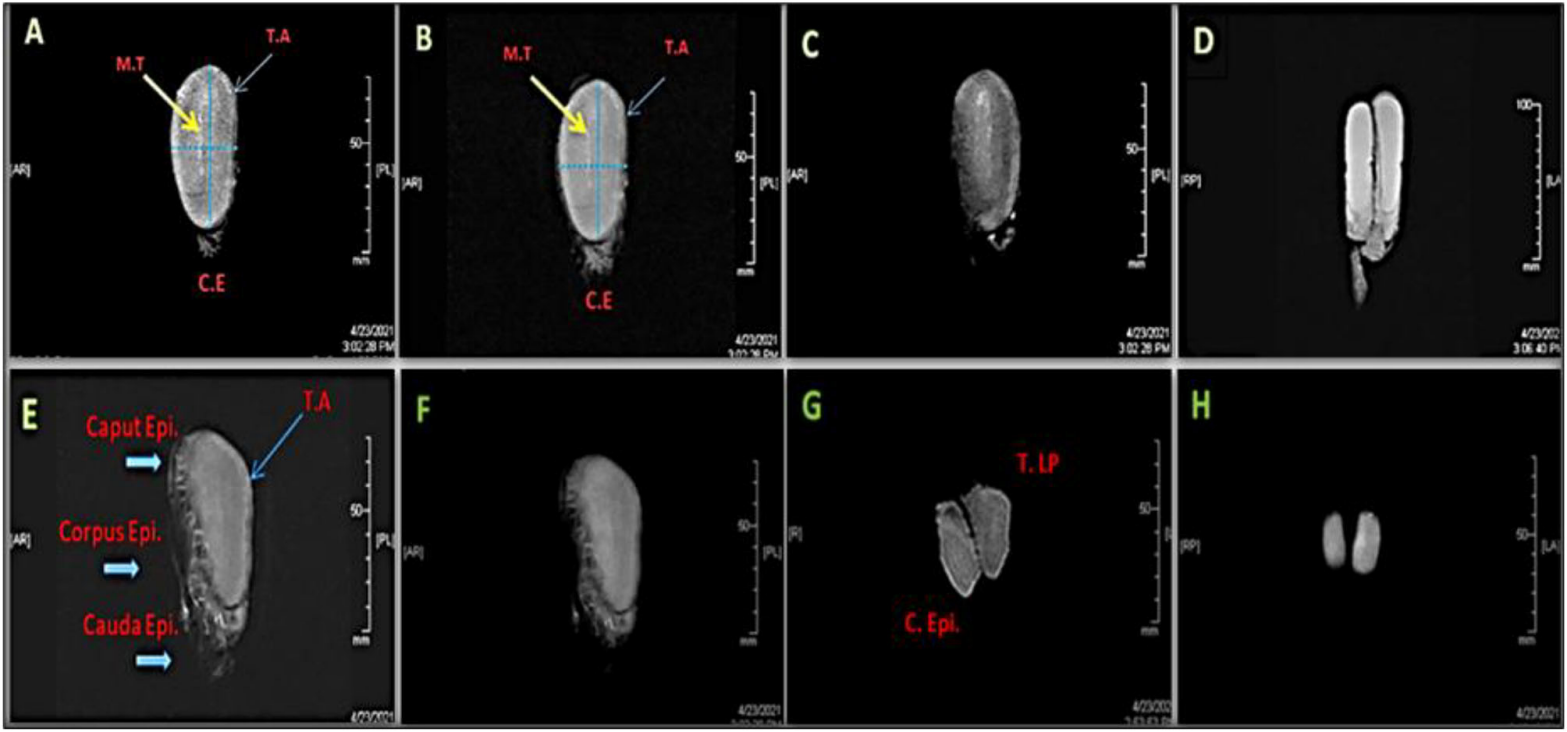
Magnetic resonance images (MRI) of a right whole camel testis **(A–E)** and epididymis (E, F) showing sagittal section scanned by different series; T1-Spin Echo MR [**(A)**: Front view; F: Lateral View], T2-Fast Spin MR **(B)**, 3D Fast-Spoiled Gradient-Echo MR **(C,D)**, 3-Plane Localizer MR **(E)**, T2 Spin-Echo of MR **(G)**, and 3-DFastSpoiled Gradient-Echo of MR **(H)**. MT: Mediastinum testis, T.A: Tunica albuginea, C.E & C. Epididymis: Cauda epididymis, T.LP: Testis lowers part.

Testicular and epididymal MRI T1-, T2-dynamic-enhanced MRI were applied for intratesticular tissue characterization compared to ultrasound scanning was shown in Figure 2. MRI results revealed that both the testes and epididymides have homogenously intermediate signal (T1) and testes have hyperintense signal, with slightly lower signal in the epididymides (T2).

### Angioarchitecture of Camel's Testicular Artery

**Figure 3:**
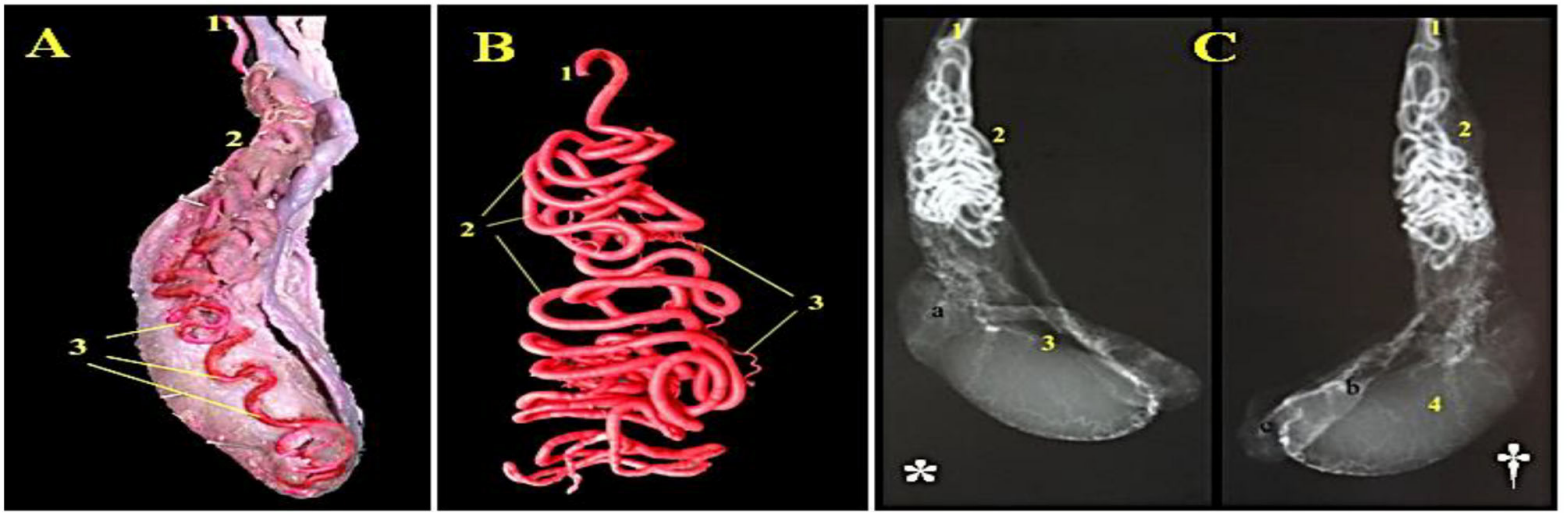
A photograph of the camel's right testis showing distribution of the testicular artery (Latex injection technique) is shown in **(A)** (1) A. testicularis, (2) Pars funicularis cum a. testicularis and (3) parsmarginalis cum A. Testicularis.A photograph of a corrosive arterial cast of the camel's testis presented the convolutions of the testicular artery (Epoxy injection technique) is shown in **(B)** (1) A. testicularis, (2) Arterial windings and (3) Rami funicularis. A radiograph of camel's testis showing distribution of the testicular artery injected with urographine (*: medial view, ^†^: lateral view: Exposure factors were 75 Cm. FFD, 30 KVP and 95 MAS) is shown in **(C)** (*a. Caput epididymidis, b. Corpus epididymidis, c. Cauda epididymidis*): (1) *A. testicularis*, (2)*Pars funicularis cum a. testicularis*, (3) *Parsmarginalis cum a. testicularis*, and (4) *Tunica arteriosa testis*.

A total of 2 testicular arteries branch from the ventrolateral aspect of the abdominal aorta and travel to the epididymides (Figure 3). Topography of that long convoluted artery is more convenient to deal with the marginal, funicular and abdominal vessels (Figures 3A,B). The course and origin of the abdominal part of the arterial vessels were traced in the abattoir. Most of examined cases present the right artery with a slight cranial level rather the left 1 (Figure 3C).

#### A. Testicularis Pars-Abdominalis cum

Abdominal part of the testicular artery is extended from the abdominal aorta up to the deep inguinal ring, obliquely leaving its origin and laterally downward over the deep surface of the psoas major M., and then crossing the ureter and external iliac artery till reaching the annulus inguinalis interna and corresponding the otherspermatic cord content. Its length was recorded as 48–55 cm. No branches were detected from the abdominal part of the testicular artery throughout the preinguinal length.

#### Pars Funicularis cum A. Testicularis

As shown in Figures 3A–C, the funicular part of testicular artery is extended from annulus inguinalis interna to extremitas capitata. Throughout the canalis inguinalis, it presented numerous loops in a spiral course and gathered forming a conical mass with its base for covering the extremitas capitata. The vascular cone is elongated in outline formed of several wide and narrow windings which are intermingled with each other. The cone bulk is formed of several arterial loops in a clockwise direction. The arterial loops are coiled repeatedly as a helical-vascular mass, and markedly increased in number reaching their maximum at the distal third of the cord. At the head of the testis, the convolutions are diminished in contrast to straightening of the main vessel. The length of the vascular cone varied between 10 to 19 cm, while the unrolled funicular part is 180–240 cm in length and 1.2–2.3 mm in diameter. The mean number of the windings arrangement is 95–125. Along its course, the funicular testicular artery gives off: A. Caput epididymalis, A. Cauda epididymalis and Rr. Funicularis.

#### Rr. Funicularis cum Pars Funicularis

About 5–6 funicular branches were emerged from the vascular cone at variable levels and depths and extended superficially supplying the cremaster muscle, tunica vaginalis and spermatic fascia (Figure 3).

#### Aa. Epididymalis

The epididymal artery is a stout vessel that arises from the pars funicularis, middle to the vascular cone, descends to the body and tail areas of epididymis to be ramified in a dichotomy fashion. It penetrates through the corpus epididymis and continues till reach the region of the tail to ramify into three branches: one of those branches is directed medially to the vas deferens to anastomose with the A. defentisbranches (Figure 3).

#### Pars Marginalis cum A. Testicularis

The marginal portion of the testicular artery along the epididymal border forms several wide helical windings (Figure 3). On reaching the extrimitase caudate of testis, it turns cranially ascending on the free portion for 2–3 cm and then terminates into 2–3 branches: cranial, medial and lateral testicular arteries. The cranial testicular branch appears as the direct continuation of the parent vessels extending proximally along the free border of the testis where it is divided into 3–4 branches that ramify in the Margo liber.

The medial or that lateral arteries turns around the extremity of the epididymal tail forming two or three of spiral coils proceeding through the dorsomedial and dorsolateral direction toward the both testicular surfaces. Both mentioned arteries are divided into 7–9 min branches of the 2nd order and extended dorsocranially in a wavy manner toward the Margo-epididymidis. Such branches are loosely arranged on both surfaces of the testes forming tunica arteriosa testis. Rami parenchymal, 12–14 in number, are delicate arteries arising from the tunica arteries along the whole surface of the testis. They independently run, without anastomoses, throughout the testicular parenchyma toward the mediastinum.

Angiographic features of camel testicular artery are shown in Figure 3 using latex injection (Figure 3A) with subsequent corrosive arterial cast (Figure 3B) and radiography (medial & lateral views) following urographine injection (Figure 3C^*^ and †).

## Discussion

Our study aimed to investigate the anatomical features of the camels' testicles and compare the characteristic benefits of both the ultrasonography (US) and magnetic resonance imaging (MRI) in detecting the histological architecture of the testicular parenchyma in dromedary camel. Our findings agreed with earlier report on the relationship between the testicular measurements and echotexture in camel (28, 29). Compared to MRI, ultrasonography is known for its versatility in detection of the changes associated with the seminiferous tubule microstructures with the same sensitivity as 3-PLANE LOC, 3D-FSPGRE, and T2FSE (10). MRI is more accurate and sensitive than US in diagnosis of some testicular pathologies (22, 23); but in testicular histomorphology, US is equally sensitive for both the fluid- and protein imaging as most MRI series (16), resulting in the observed correlations between the quantitative properties of the images, the cell density, and the seminiferous tubule area. The MRI showed higher number of significant correlations with histological variables that may be attributed to the good resolution of the produced image (21, 30); however, it was not good enough to give significant correlation with the cell density.

The present findings confirmed the proficiency of US and MRI in evaluating the histomorphological parameters of testicular parenchyma. However, the later method presented more validation of the different clinical or *in situ* applications, either. However, vasculature and innervation may affect the sensitivity of both techniques in detecting the testicular changes and histomorphology. More studies are required to validate the capability of MRI series in examining the testicular tissue architecture. The vascularity pattern of testis had species variations of lengths of spermatic cord as well as the size and position of the testicles (31). This study, and that recorded before (32), confirmed the origin of the testicular artery in camel from the ventral or ventrolateral aspect of the abdominal aorta. Generally, the genital gland artery arises before the caudal mesenteric artery at the level between the 4th and 5th lumbar vertebrae. In all examined camels, this testicular artery showed no duplication with the other arterial branches. There was no significant difference between Vernier caliper, US and MRI in the morphometric of testes such as length, width, depth, and volume. This means that evaluating testicular morphometric can be done efficiently using Vernier caliper, US or MRI with no significant difference. While, only US and MRI can be used in detecting the histological architecture of the testicular parenchyma.

The coiling pattern and degree of tortuosity of the testicular arteries were also clarified as formed of numerous loops disposed in the spermatic cord, the same pattern as reported in a previous study (33). The parent vessel was convoluted repeatedly crossing the spermatic cord, emphasizing that the greater coils number and the associated slow velocity of blood flow result from the long vessels have the ability to efficiently support the heat exchange surrounding the entire testicular tissue. Similar explanations were given by Hofmann (34) and Hess et al. (35) in cattle bull, Osman et al. (33) in camel, and Elgaafary et al. (36) in the buffalo bull. The degree of arterial convolution in the spermatic cord is directly proportional to the size of the testis which explains the less tortuosity in rodents, carnivores, and human which all have the small sized gonads, and mostly straight testicular artery (31).

The finding reported in a previous study that one epididymal artery was detected instead of two arteries (33) agreed with ours. However, a controversial result was reported by El-Gaafary and Aly (37) who recorded two epididymal arteries. El-Gaafary and Aly (37) stated that pars marginalis of the testicular artery is terminated near the tail extremity giving two branches. On the other hand, we detected three terminal branches for the pars marginalis, which disagreed with previous studies which roughly stated 3–4 (32) or 2–4 (33) branches of pars marginalis.

## Conclusions

There is a potential application for both US and MRI to thoroughly examine the histomorphological characteristics of the camels' testes and hence monitor the male reproductive fertility. However, US can be the primary technique of imaging for assessment of the male genital health due to its proficiency and the lower cost. However, MRI is beneficial when the sonograms are inconclusive or equivocal. MRI shows the examined tissues in greater anatomical details compared to ultra-sonography. Further studies are needed to compare between characteristics of US and MRI of normal testes and epididymis with testicular artery angiography in living camel during rut season and non-rut season and between normal healthy and affected diseased genitalia. Indeed, using these innovated imaging techniques specially IMR on usual clinical practice is still not commonly adopted; while, they might be restricted to some dedicated research centers.

## Data Availability Statement

The raw data supporting the conclusions of this article will be made available by the authors, without undue reservation.

## Author Contributions

RS, KK, RM, and AA prepared conception and design of study and performed data curation and interpretation of data. HH, RE, and AA manipulated and statistically analyzed the data and drafted the manuscript. RS, HS, MA, AS, and AA carried out final writing, critical review, and revision. All authors have read and approved the final manuscript.

## Conflict of Interest

The authors declare that the research was conducted in the absence of any commercial or financial relationships that could be construed as a potential conflict of interest.

## Publisher's Note

All claims expressed in this article are solely those of the authors and do not necessarily represent those of their affiliated organizations, or those of the publisher, the editors and the reviewers. Any product that may be evaluated in this article, or claim that may be made by its manufacturer, is not guaranteed or endorsed by the publisher.
